# Car engine sounds recognition based on deformable feature map residual network

**DOI:** 10.1038/s41598-022-06818-z

**Published:** 2022-02-17

**Authors:** Zhuangwen Wu, Zhiping Wan, Dongdong Ge, Ludan Pan

**Affiliations:** 1 Zhejiang Industry Polytechnic College, Shaoxing, 312000 China; 2 Society of Automotive Engineers of Shaoxing, Shaoxing, 312000 China; 3 Shaoxing Sci-Tech Museum, Shaoxing, 312000 China

**Keywords:** Information technology, Information theory and computation

## Abstract

Aiming at the difficulty in extracting the features of time–frequency images for the recognition of car engine sounds, we propose a method to recognize them based on a deformable feature map residual network. A deformable feature map residual block includes offset and convolutional layers. The offset layers shift the pixels of the input feature map. The shifted feature map is superimposed on the feature map extracted by the convolutional layers through shortcut connections to concentrate the network to the sampling in the region of interest, and to transmit the information of the offset feature map to the lower network. Then, a deformable convolution residual network is designed, and the features extracted through this network are fused with the Mel frequency cepstral coefficients of car engine sounds. After recalibration by the squeeze and excitation block, the fused results are fed into the fully connected layer for classification. Experiments on a car engine sound dataset show that the accuracy of the proposed method is 84.28%. Compared with the existing state-of-the-art methods, in terms of the accuracy of recognizing car engine sounds under various operating conditions, the proposed method represents an improvement over the method based on dictionary learning and a convolutional neural network.

## Introduction

### Motivation

Car engine sound refers to the sound produced by a car engine when it is being started and when it is running; it provides information for assessing the performance of the car and the engine^1^. For example, certain sounds in the engine’s combustion chamber, such as the vibration of the engine body, the sound of detonation of the gas mixture, the sound of a spark plug jumping, the sound of a valve lifting and seating, the scraping sound of piston rings, and the friction of the crankshaft mechanism, can help one to judge the operating condition of the cylinder. Sounds generated by other engine parts, such as the transmission belt, exhaust, cooling fan, air conditioner pump, and generator, can help one to determine whether the engine is running correctly. Typical car engine sounds include those made during the operating conditions of starting, idling, acceleration, rapid acceleration, and deceleration. These sounds cover a wide energy distribution and frequency range, and the frequency changes with time, often containing background noise. It is therefore difficult to extract the features and recognize the sound of a typical car engine. Research in this area can provide better processing methods for relevant sound signals and improve the diagnosis of car performance as well as malfunction.

### Previous studies

Substantial research has been performed on the recognition of car engine sounds^1–4^. Thomas and Wilkins^1^ used cepstrum analysis to identify engine sounds from noise. Kemalkar and Bairagi^2^ used a Mel frequency cepstral coefficient (MFCC) algorithm to extract sets of engine sound features from acoustic signals collected by engine sensors. The signals were then used to diagnose engine faults, and this improved the success rate. de Oliveira et al.^3^ studied the impact of engine noise excitation on the sound quality of a vehicle, and adopted a speed feedback control strategy to reduce engine loudness and improve the overall perception of engine sound. Wang, Ma, Zhu, Liu and Zhao^4^ proposed a noise-based engine malfunction diagnostic method based on a Hilbert-Huang transform (HHT) and support vector machine (SVM); the method has successfully processed stationary and non-stationary signals. However, our study is more challenging because the engine sound studied has more components, and the signal features and background noise are more complex.

The convolutional neural network (CNN) has become an effective means of processing sound signals^5,6^; it offers significant advantages in artificial modeling, feature extraction, recognition performance, and reduced processing time. However, training a CNN requires a large number of labeled samples; otherwise, the performance of the network will be limited. Due to the small sample of engine sounds involved in this study, direct CNN processing is not applicable. Research^7^ shows that the structure of the time–frequency image can be used to reveal the essential characteristics of the sound signal, and that it is an acoustic parameter that can reflect the time domain and spatial domain structure of the sound signal. In addition, the time–frequency image of a sound shows a dynamic process that changes with time, which can quantify the background noise with more frequency components to a specific region and reduce the interference of background noise on the sound signal^8^. Therefore, we can convert the engine sound to a time–frequency image, and use ImageNet^9^ to pre-train a CNN. Following the migration learning strategy, the parameters of the pre-trained network are transferred to the trained network of the engine sound time–frequency image^10^. The depth of the network has a significant impact on CNN performance^11^. Khamparia et al.^12^ showed that a deep network structure is more suitable for feature extraction from the time–frequency image of the sound signal, but an improper network structure can degrade performance. He, Zhang, Ren and Sun^13^ proposed a residual network (ResNet) to solve the problem of extracting features from natural images. Wiatowski and Bölcskei^14^ pointed out that the regular convolution kernel in a traditional CNN often samples areas of the image that are not of interest, and that its feature extraction ability is low. The deformable convolution network (DCN) proposed by Dai et al.^15^ can, to some extent, solve that problem, but the DCN has more parameters and can only be used in the current network layer.

### Contribution

The logarithmic Mel spectrogram^16,17^ is an expression of the time–frequency image of a sound signal that can better display its energy distribution and time–frequency characteristics. Our analysis of the logarithmic Mel spectrogram of car engine sounds shows that the energy distribution of an engine sound signal extends over a wide frequency range. Compared with natural images, the logarithmic Mel spectrogram is richer in texture, and the region where the signal energy is concentrated has a more complex geometric structure and edges that are more irregular. For this reason, we propose a deformable feature map residual network to identify the logarithmic Mel spectrogram of engine sounds. The designed deformable feature map residual block (DFMRB) changes the positions of the pixels in the feature map and obtains a shifted feature map by adding an offset variable to the logarithmic Mel spectrogram. A two-layer convolution extracts the features of the shifted feature map. We superimpose the shifted feature map with the feature map extracted by the convolutional layer through a shortcut connection and use it as the output of the block. The designed convolution kernel can change the shapes and positions of the actual sampled pixels according to the logarithmic Mel spectrogram of the engine sound, so the network can focus on sampling the region of interest in the feature map and improve the feature extraction ability. We also perform an MFCC fusion of the features extracted by the deformable feature map residual network with the original engine sound signal and recalibrate with the squeeze and excitation block (SEB)^18^ to further improve the feature description capability of the network.

The engine sound recognition technology studied in this paper is a non-speech-recognition technology that provides a realistic case for the study of machine language on the sound mode of mechanical systems. The research results of this paper can serve as a reference in the application of artificial intelligence for monitoring automobile sound and in the use of deep learning technology for diagnosing automobile malfunctions, so as to predict potential car problems in advance. This is especially applicable to self-driving cars.

## Methods

*Analysis of time–frequency features of five categories of engine sound in typical operating conditions*. The logarithmic Mel spectrum of a signal can be obtained from

1 $$M = \log H\left( k \right)\left| {\mathop \sum \limits_{n = 0}^{N - 1} x\left( n \right)w\left( n \right)e^{{\frac{ - 2\pi ikn}{N}}} } \right|^{2} ,$$

where *k* = 0, 1, …, *N* − 1, *M* is the logarithmic Mel spectrogram matrix of the signal obtained from a superposition of the logarithmic Mel spectral vector of each frame of the signal, *H*(*k*) is the Mel filter set function, *x*(*n*) is the input sound signal, *w*(*n*) is the Hamming window function, and *N* is the length of the Hamming window^18^.

**Figure 1:**
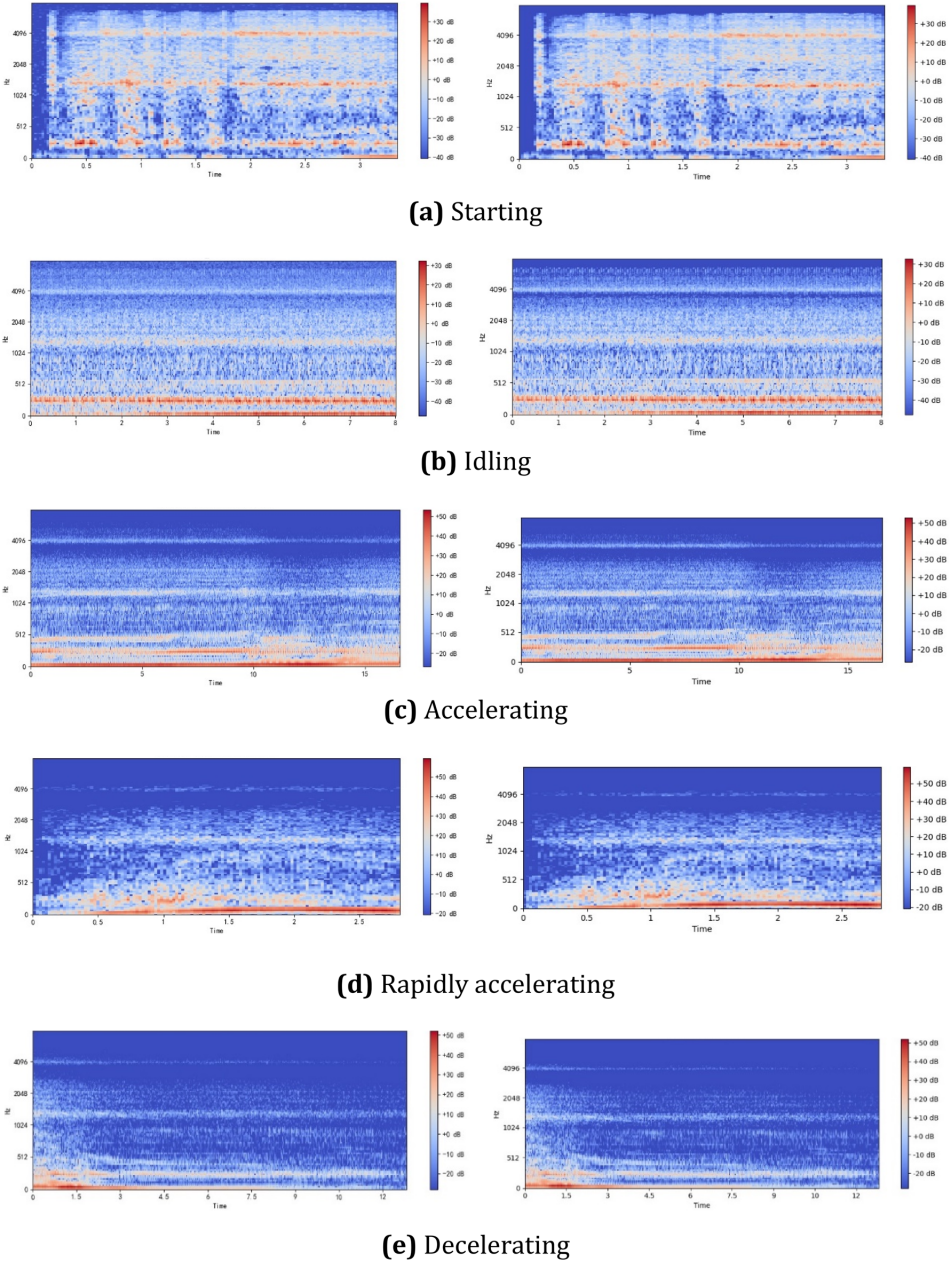
Logarithmic Mel spectrogram of engine sound with different signal-to-noise rations (SNRs) under five typical engine operating conditions.

The signal-to-noise ratio (SNR) of an acquired car engine sound is unknown, but can usually be considered high or low. Based on Eq. (1), the logarithmic Mel spectrogram of the five categories of sound of a car engine in operation can be obtained, as shown in Fig. 1, where the left and right graphs in the insets show signals with high and low SNRs, respectively. Although the SNR may differ, the logarithmic Mel spectrogram of a car engine sound generally has a similar horizontal stripe-like texture to a certain extent, as shown in Fig. 1b for the idling operating condition, and Fig. 1c for the accelerating operating condition. Horizontal stripes of variable width indicate that the frequency components of the sound change over time, while vertical stripes of variable width imply that frequency components have different durations. The logarithmic Mel spectrogram of a sound with a lower SNR has a richer texture than that with a high SNR. The logarithmic Mel spectrogram of the starting operating condition in Fig. 1a shows similar stripe textures in the left and right graphs. The image of this operating condition is brighter than that of other operating conditions, which indicates that it has higher-energy components for the corresponding frequencies. It can be seen from the logarithmic Mel spectrogram of the sound of the idling operating condition, shown in Fig. 1b, that it has a greater area of bright color, indicating that the sound of this operating condition has rich frequency components with a broad distribution of energy. The spectrum has rich texture, and the structure of the region with a concentration of energy is complex, which is the main reason that the frequency components of the engine sound change significantly with time. The logarithmic Mel spectrogram of the acceleration operating condition, shown in Fig. 1d, contains prominent curves with high grayscale values, indicating that the sound energy for this operating condition is highly concentrated on only a few frequency components. Figure 1e shows the logarithmic Mel spectrogram of the sound of the deceleration operating condition. The energy is concentrated at certain regions where the color is bright, but the color fades into dark gray toward the boundary, where the edge becomes irregular. This indicates that the energy decay of frequency components occurs at varying rates. In addition, the length of the time window and the size of the sliding step can affect the conversion from engine sound to logarithmic Mel spectrogram, but because the time window is small and the sliding distance short, the differences in the Mel spectrogram are small.

### Deformable feature map residual network

**Figure 2:**
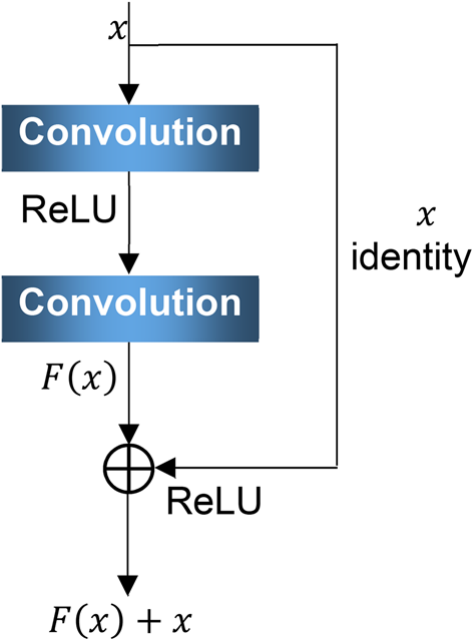
Structural diagram of residual block.

*Problems in extracting logarithmic Mel spectrogram features from engine sound using ResNet.* The CNN has demonstrated excellent performance in image processing^19^. ResNet^13^ is the classic structure of a CNN, and it can solve the problem of rapidly declining performance as the depth of a CNN increases, and is efficient at natural image feature extraction. ResNet is formed by stacking residual blocks, whose structure is shown in Fig. 2, where *x* is the input of the residual block, identity *x* is known as the shortcut connect, convolution refers to the convolutional layer, and *F*(*x*) is the output of *x* after two layers of convolution. After being superimposed with the shortcut connect, *F*(*x*) outputs *F*(*x*) + *x.* The feature map output *F*(*x*) + *x* by the residual block has the same size as the input, and the residual block acquires a stronger learning ability by fitting the residue between the input and output with two convolutional layers.

**Figure 3:**
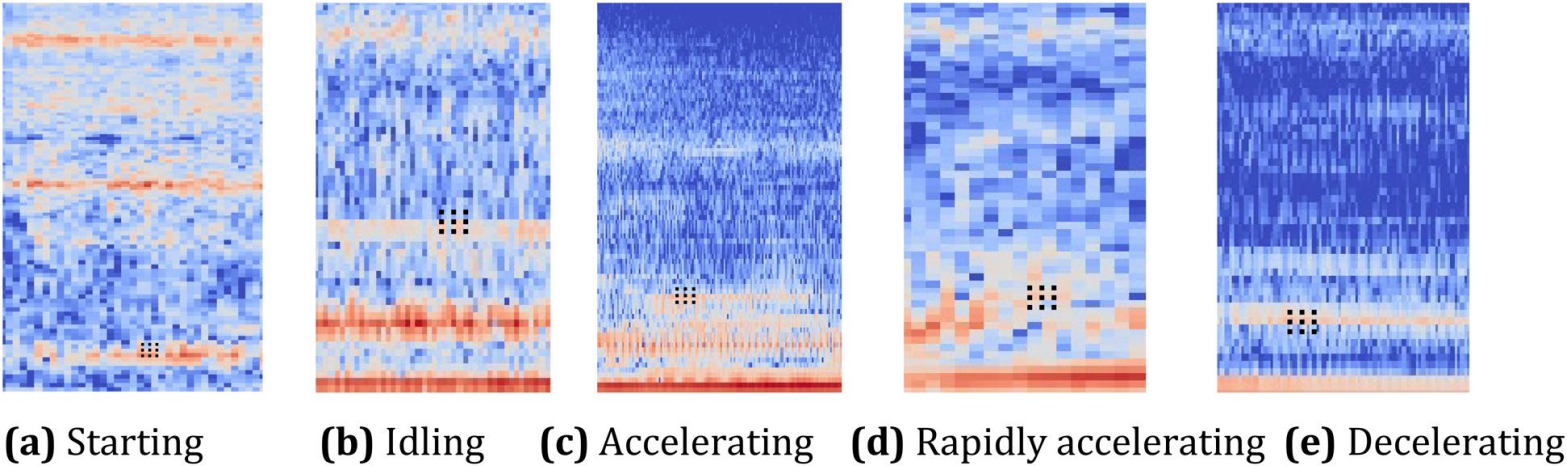
Conventional convolution sampling positions in logarithmic Mel spectrogram.

Because the engine sound is affected by the interference of background noise, the region where the logarithmic Mel spectral energy is concentrated contains information about the target sound as well as background noise. This complicates the geometric structure of the region on the logarithmic Mel spectrogram, makes the edge of the profile even more irregular, enriches the spectral stripes of sound of different operating conditions, and decreases their differences. However, since the size, shape, and sampling positions of the convolution kernel of the residual block are fixed (see Fig. 3), such fixed convolution kernel units often cannot concentrate on the regions of interest when extracting logarithmic Mel spectral features of the engine sound. For this reason, the traditional ResNet method for extracting features of natural images cannot properly process the feature extraction problem of engine sound. In Fig. 3, the black dots represent the positions of sampling pixels of the traditional convolution kernel. Because the shape of the traditional convolution kernel sampling points is a fixed rectangle, so is the shape of the sampled pixels. The pixels at the centers of rows 1 and 2 in Fig. 3a are in relatively dark regions, and only the pixels in the second and third rows in Fig. 3b are in bright regions. Similarly, sampling pixels in Fig. 3c–e can easily fall in regions of small grayscale value and have small changes. These regions are dark, and the signals have extremely low frequency content, so the time–frequency characteristics of the signal cannot be displayed. This implies that the traditional convolution kernel will sample in regions of little consequence or interest. The extracted feature signals will be low, and the descriptive ability of the network will be poor.

#### DFMRB

Deformable convolution is a better kind of convolution for extracting features of images^15^. It is formed by adding an offset variable to the position of each sampling point of the convolution kernel. This allows arbitrary sampling near the current position, so that the sampling is no longer limited to a regular grid of an ordinary convolution kernel.

**Figure 4:**
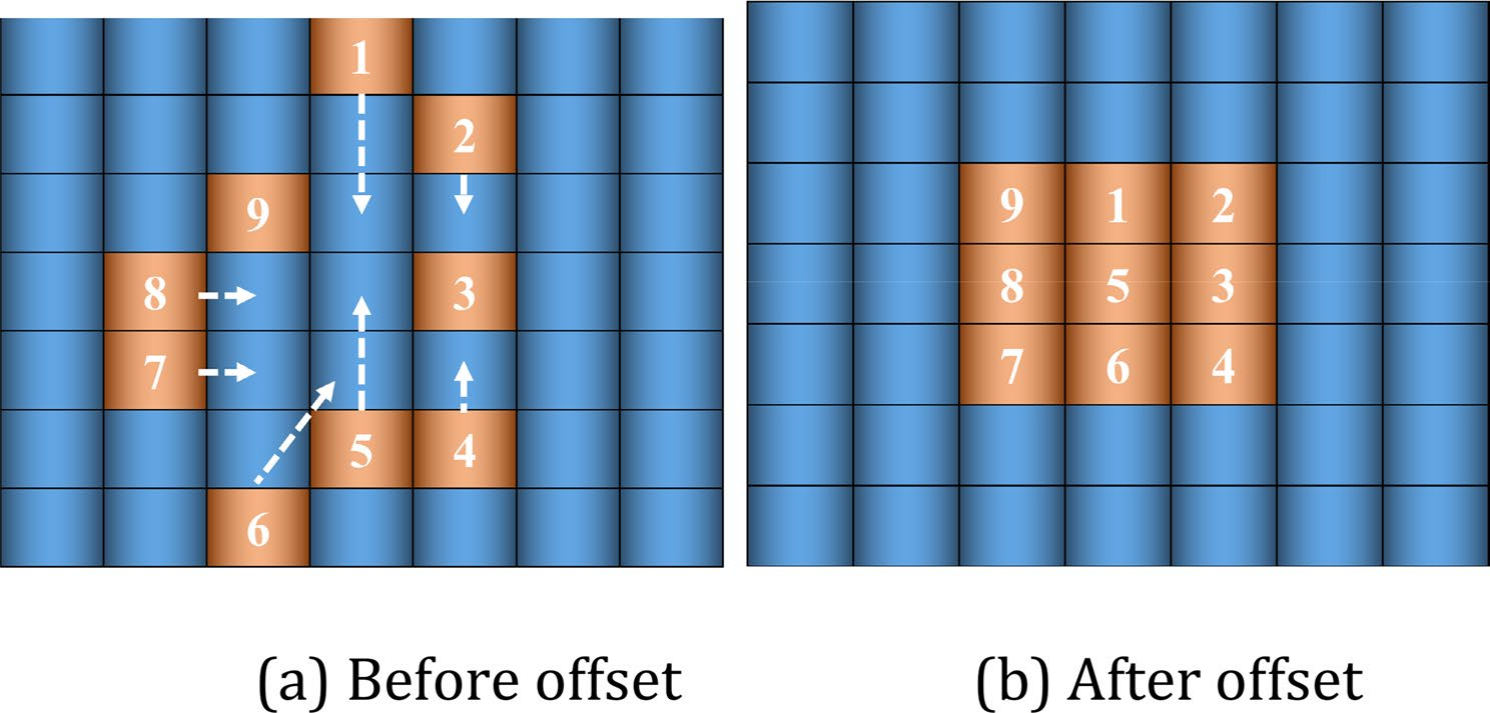
Feature map pixel offset in DFMRB.

Based on deformable convolution, we designed DFMRB, as shown in Fig. 4. Pixels are shifted by adding an offset variable to the feature map, and a shortcut connect superimposes the shifted feature map with the feature map extracted by the subsequent convolutional layer. This avoids the limitations of the fixed convolution kernel, allows the network to arbitrarily sample features in the vicinity of the current position, and ensures that the information of the shifted feature map can be transmitted to the lower network layer, so that the network can sample important areas or regions of interest in the spectrogram, and enhance the feature-extraction capability of the logarithmic Mel spectrogram.

Each square in Fig. 4a represents a pixel in a specific position of the feature map. The small, dark-blue squares represent the sampling range of traditional convolution, serial numbers 1–9 are pixels of the region of interest, and the arrows point to the direction of the offset displacement. The direction and distance of the pixel point displacement in the feature map are obtained by network training. When the value of "loss" drops to the point where the convolutional layer can sample the features of interest, the pixels of interest are concentrated in the perceived field of view. Therefore, when the offset of the feature map pixels increases, the pixels in the traditional convolution sampling range will change accordingly until the pixels of interest are sampled (such as pixels 1–9). The shifted feature map is shown in Fig. 4b.

**Figure 5:**
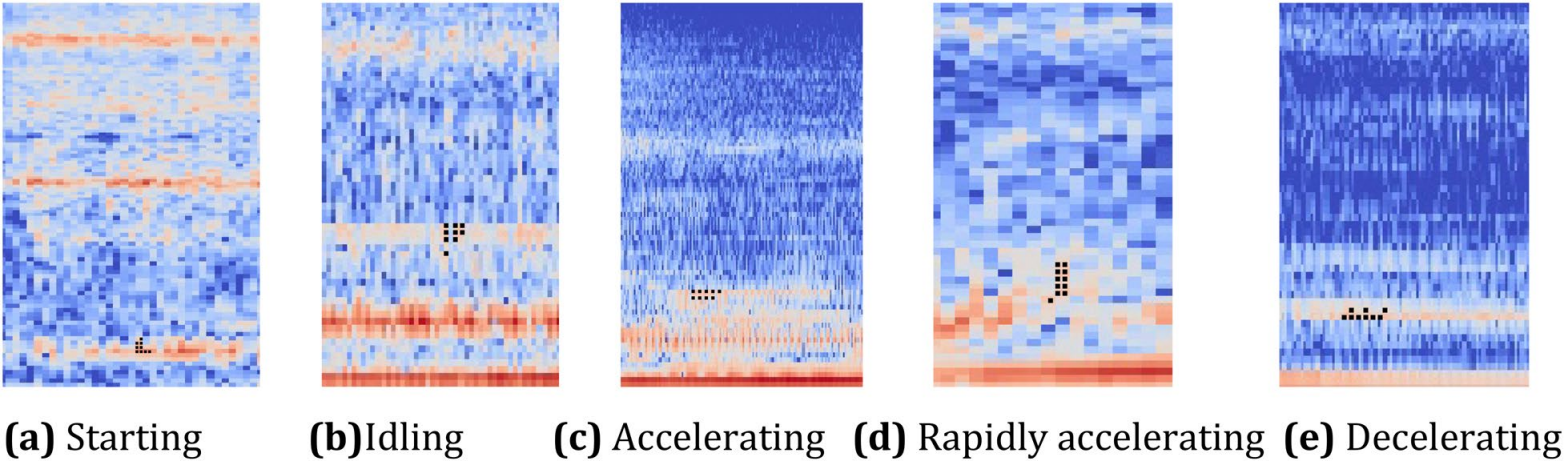
Sampling position of DFMRB in logarithmic Mel spectrogram.

DFMRB is used to extract the features of the logarithmic Mel spectrogram of engine sound. The convolution sampling position is shown in Fig. 5, in which the black dots represent the actual positions of convolution kernel sampling pixels. Although the shape of the sampling points of the convolution kernel is still rectangular, because the pixels of the feature map are shifted, the pixels sampled by the convolution kernel have also changed. The optimum pixels sampled by the network-trained convolution kernel are always located in a bright region; therefore, with the offset added to the feature map, the network-trained lower network layers can concentrate on sampling from regions of interest.

**Figure 6:**
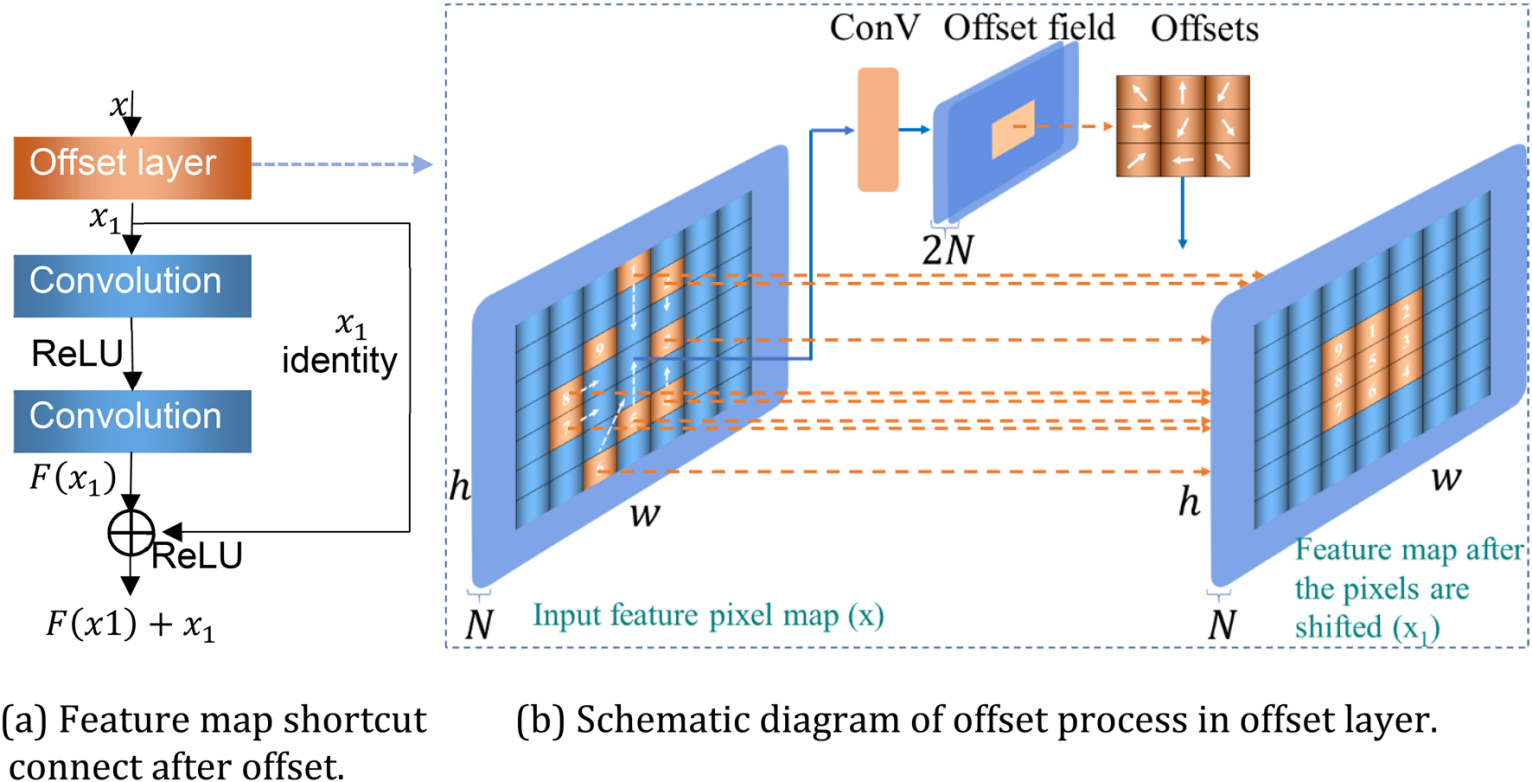
Diagram of structure of DFMRB.

The shortcut connects designed in this paper to transfer the shifted feature map information to the lower-level network are shown in Fig. 6a, and the offset process is shown in Fig. 6b, in which the first layer is a DFMRB offset layer for implementing pixel displacement in the feature map, *x* is the input feature map, *N* is the number of channels, and *w* and *h* are respectively the width and height of *x.* The size of *x* remains unchanged after the convolutional layer, and the offset layer can learn the offset field of 2* N* through a two-dimensional convolutional layer. Because the pixel offset of the feature map contains two directions, vertical and horizontal, the number of channels of the offset field is twice that of *x.* The values of every two channels in the offset field constitute the offset of one channel of *x*. The implementation is as follows. The parameters of the first *N* channels of the offset field are used as the horizontal displacement distance of feature map pixels, and the parameters of the remaining *N* channels are used as the vertical displacement variable. The parameter on the *n*th channel and the parameter on the *n* + *N*th channel constitute the offset variables of a given feature image pixel. The values of the offset variables are then added to the index value *x*_*i*_ of pixel *x* to obtain the new index values after the offset and to restrict all the index values within the dimension of the feature map. Because the index values of the current positions may not be integers, the indexed pixel value must be determined using bilinear interpolation. This replaces the pixel value corresponding to the position of the original index value, thereby obtaining the feature map *x*_1_ after the pixel offset. The index value keeps changing as the offset layer parameters are updated. In this manner, the pixels gradually shift in the optimum direction and with the optimum distance, with pixel *x* shifting toward *x*_1_ and reaching the goal of pixel sampling in the region of interest by the lower-layer network.

In Fig. 6, *x*_1_ produces output *F*(*x*_1_) after two convolutional layers and is directly superimposed with *x*_1_ to produce output *F*(*x*_1_) + *x*_1_*.* To implement the transmission of the information in the offset feature map, we place the original residual block behind DFMRB to produce a block output of $F(F(x_{1} ) + x_{1} ) + F(x_{1} ) + x_{1}$. This output retains *x*_1_ and passes it to a lower-level network so that it can still focus the sampling on a region of interest. If the lower-level network is also a residual block, then multiple residual blocks can share the offset feature map information.

**Figure 7:**
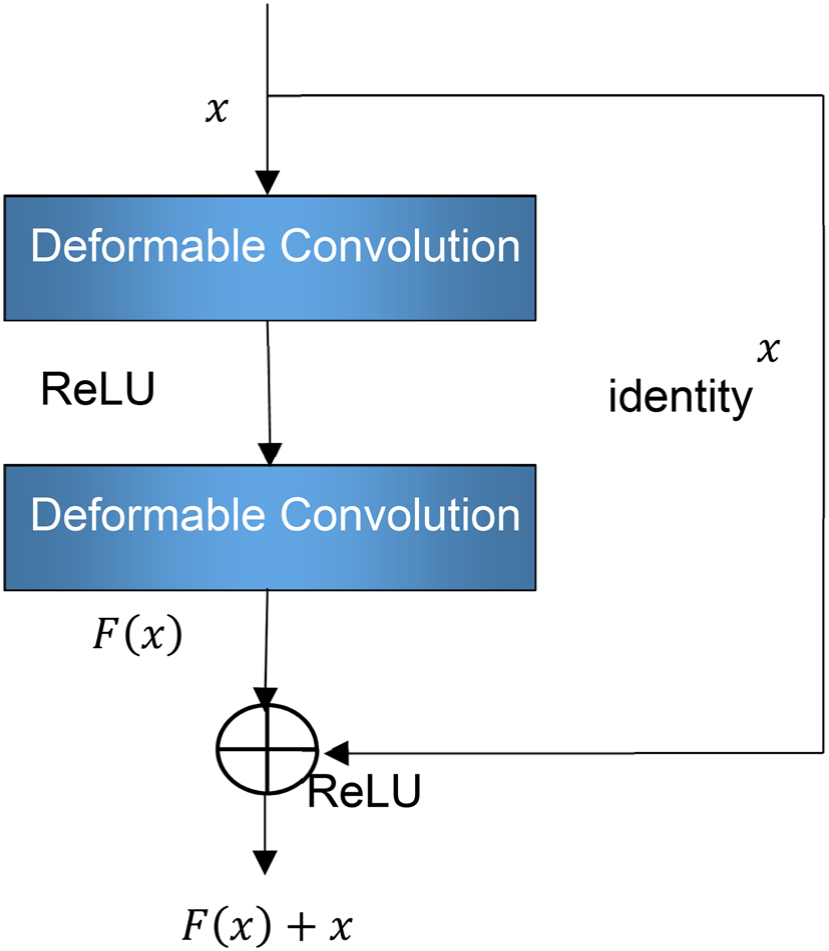
Diagram of adding deformable convolution to residual network.

When the deformable convolution^15^ is added directly to ResNet, the deformable convolutional layer will directly replace the traditional convolutional layer in the residual block to form a deformable convolution residual block, as shown in Fig. 7. Deformable convolution can be added to ResNet only as a single network layer, affecting the sampling in only the current network layer, and the lower network layer of ResNet still cannot concentrate the sampling in a region of interest to the input feature map. Replacing more convolutional layers with deformable convolutional layers will substantially increase the offset variable computation. DFMRB is implemented by adding an offset layer before the shortcut connect, so the number of offset parameters is only half that of Dai et al.^15^. In addition, DFMRB changes the positions of pixels in the feature map and separates the pixel offset from the convolution process. The feature map information of the shifted pixel can still be passed to the next module, and the lower-level network can still concentrate the sampling on the region of interest, further reducing the number of parameters to be calculated.

#### Deformable feature map residual network structure

**Figure 8:**
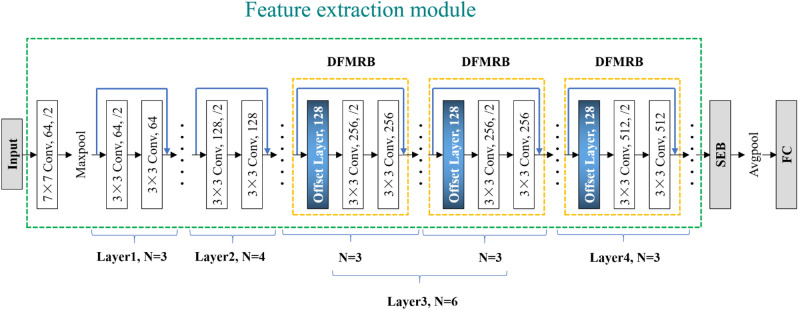
Diagram of deformable feature map residual network.

Figure 8 shows the deformable feature map residual network designed in this paper based on DFMRB. The network consists of three modules: feature extraction, SEB, and classification. The feature extraction module includes 16 residual blocks, and the low-level network is composed of seven ordinary residual blocks, which are used to extract the texture features of the engine sound images for five operating conditions. The high-level network contains three DFMRBs to extract the semantic features from images of engine sounds. Each DFMRB is followed by ordinary residual blocks with one offset feature map and two shared feature maps in order to improve the modeling ability for semantic features of engine sound. After the extracted features are recalibrated by SEB, feature classification is carried out through an average pooling (Avgpool) process and by output to the fully connected (FC) layer.

### Experimental

There are many engine sounds due to different models, operating conditions, and component malfunctions. Different operating conditions have different numbers of sound samples, which has often led CNN to misidentify sounds. Due to the small number of available tagged engine sound samples, overfitting often occurs during sound recognition. We have tried to solve this problem with pitch shift^5^ balance and by increasing the number of engine sound samples.

**Figure 9:**
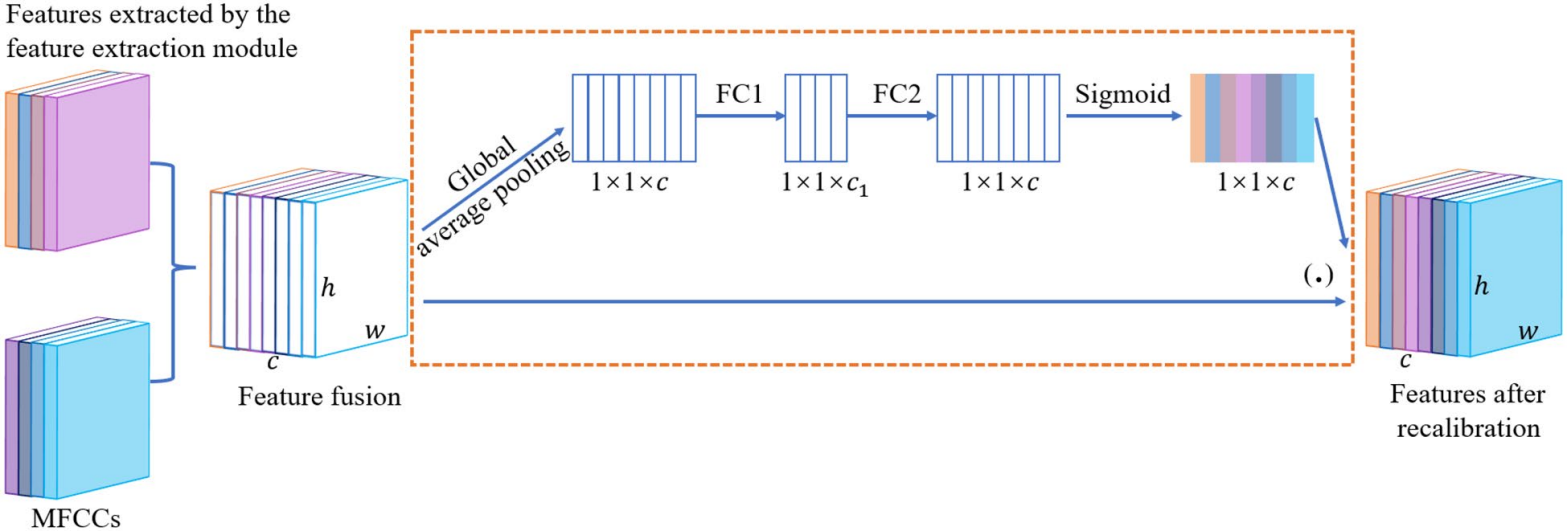
Schematic diagram of SEB used for feature recalibration.

**Figure 10:**
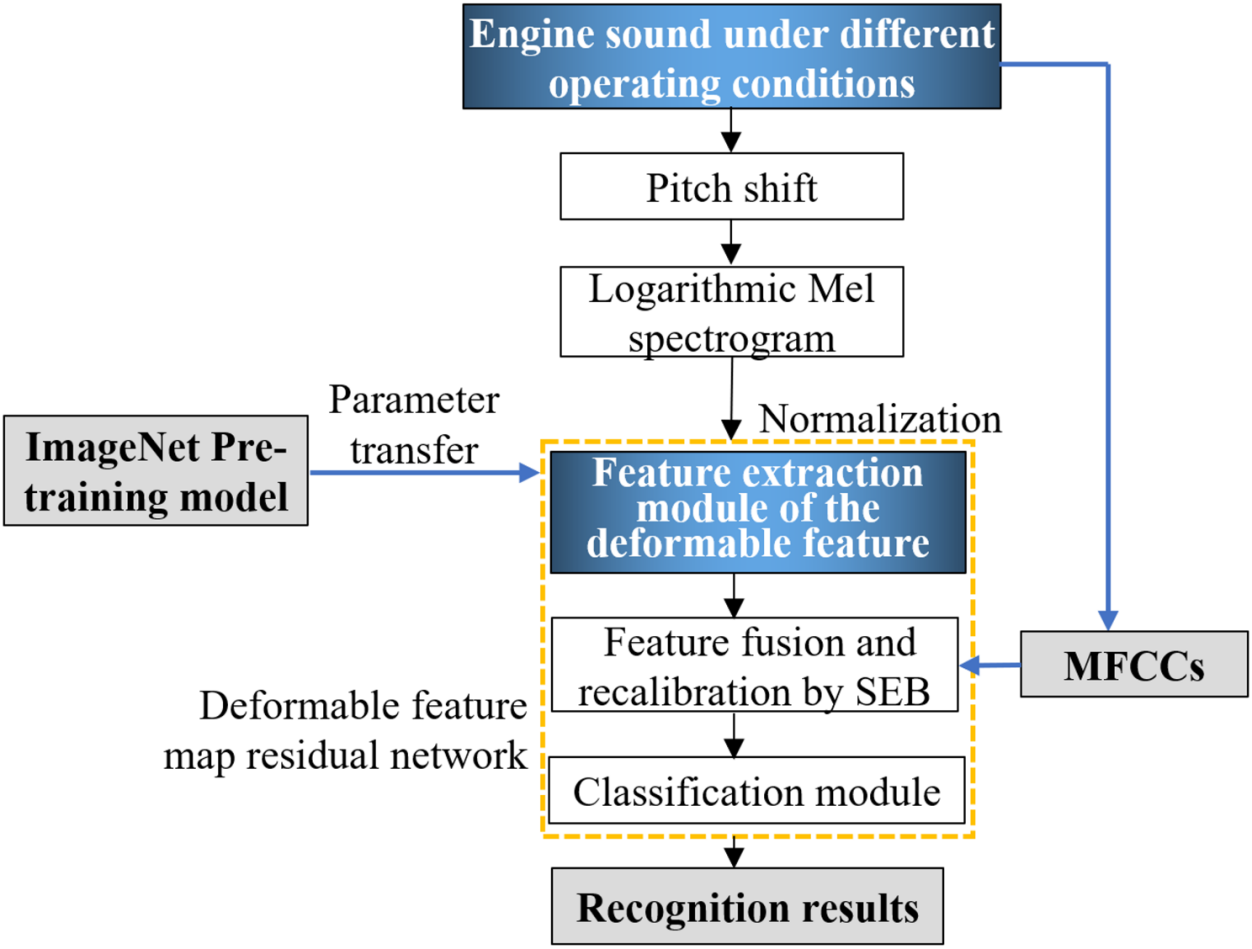
Flowchart of engine sound recognition using deformable feature map residual network.

Because there are insufficient sound samples in the engine sound database, we performed CNN pre-training with ImageNet, and transferred the obtained model parameters to the network of this paper to initialize CNN. This is an effective method for solving the problem of small number of samples in network training^20^. To further improve the ability of the network to describe engine sound features, we combined the features extracted by the deformable feature map residual network with the MFCCs of the original engine sound (signal). The fusion feature is the sum of the products of channel features and their corresponding weights, i.e., $F_{f} = \sum\nolimits_{i = 1}^{n} {a_{i} f_{i} }$, where *F*_*f*_ represents the fusion feature, *n* is the number of features to be fused, *a*_*i*_ represents the feature of each channel, and *f*_i_ represents the weight corresponding to each channel feature. The weights are automatically obtained using SEB^17^, as shown in Fig. 9, in which *c* is the number of feature channels, and *w* and *h* are the width and height, respectively, of the feature map. The fusion features are compressed by global average pooling. The two-dimensional feature of each channel becomes a real number that characterizes the global distribution of the response on the feature channel. The correlations of different channel features are fitted with the two fully-connected layers FC1 and FC2, and values in the 0–1 range are generated using the sigmoid function. These are used as weights for channel features, multiplied by them and added to the previous feature to complete the recalibration of the original fusion feature. Figure 10 shows the recognition process flowchart for engine sounds under different operating conditions using the proposed deformable feature map residual network.

## Results and discussion

### Dataset and experimental environment

 The experimental engine sound dataset used in this study was constructed from the "engine" class data in the AudioSet audio frequency dataset^21^ and the "car engine" class data in the VGGSound dataset^22^. The AudioSet audio frequency dataset is a large-scale open dataset that contains 632 audio categories and 2,084,320 manually marked sound clips (including 527 tags), each 10 s long. VGGSound is a multi-modal single-label audio dataset with 310 audio data categories and more than 210,000 videos related to sound scenes. Based on the manual tags in the AudioSet and the video scenes in VGGSound, we randomly selected a number of operating conditions samples (scenarios) of start, idle, acceleration, rapid acceleration, and deceleration in the automobile category. This is summarized in Table 1. Because the larger the sample set, the better the learning effect, we combined the samples of the AudioSet dataset and the VGGSound dataset under the same operating conditions to form the engine sound dataset used in the subsequent comparison experiments in this article.

**Table 1:** Distribution of "engine" class data samples in AudioSet and VGGSound datasets.

Engine operating conditions	Number of samples in AudioSet	Number of samples in VGGSound	Number of samples in the engine sound database used in the experiment
Starting	5490	491	5981
Idling	10,366	370	10,736
Accelerating	8692	758	9450
Rapidly accelerating	3733	232	3965
Decelerating	1810	/	1810

The experimental software environment was PyTorch 1.1.0 and Python 3.7.3, and the hardware environment was the Nvidia GTX 1080 Ti GPU. During network training, the input network image size was 3 × 224 × 224, the basic learning rate was 0.0001, the number of training iterations was 10 epochs, and the optimizer was AdaBound^23^. The experimental evaluation index was the accuracy rate, i.e., the ratio of the sum of the number of correctly classified samples to the total number of samples in the engine sound dataset. The evaluation method was tenfold cross-validation, i.e., the dataset was divided into 10 folds, nine of them taking turns as training data, and one used as test data.

### Comparison experiment of logarithmic Mel spectrogram with different parameters

**Figure 11:**
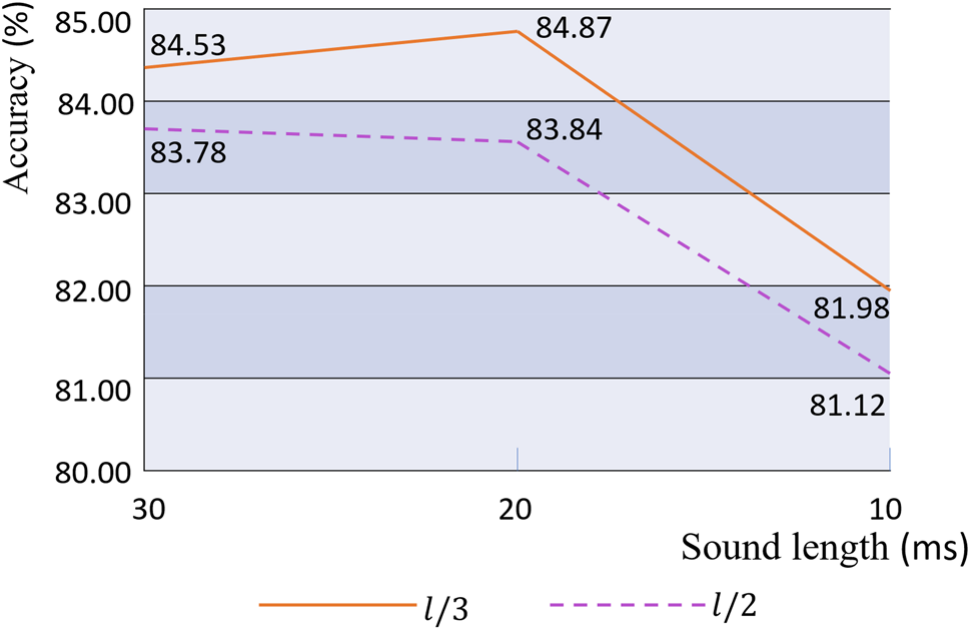
Accuracy of engine sound logarithmic Mel spectrogram under different parameters.

Engine sounds are non-stationary signals. The lengthof the time window *l* is defined as the product of the sound length and the sampling frequency. To assess the effect of different time window parameters on the classification of engine sounds, the experiment was truncated at the lengths of 10, 20, and 30 ms, and the truncated signal could be regarded as a stationary signal^24^. The sliding step length of the window function determines the time-domain resolution of the short-time Fourier transform; experiments were carried out with sliding steps of *l/*2 and *l/*3. The classification results of the logarithmic Mel spectrogram of engine sound with different parameters are shown in Fig. 11, in which dotted and solid lines represent window function sliding steps of *l/*2 and *l/*3, respectively.

Figure 11 shows that, because the shorter the sliding step, the higher the accuracy, for the same truncation length, the classification performance for engine sound for a window function sliding step of *l/*3 is better than for a sliding step of *l/*2. The accuracy was the poorest at a truncation length of 10 ms. The classification accuracy at a truncation length of 20 ms was better than at a truncation length of 30 ms. The accuracy rate was the highest at a sliding step of *l/*3 and a truncation length of 20 ms. We therefore chose a truncation length of 20 ms when converting the engine sound to a logarithmic Mel spectrogram. The time window was a Hamming window with a sliding step of *l/*3.

### Comparison experiment of data enhancement methods

**Figure 12:**
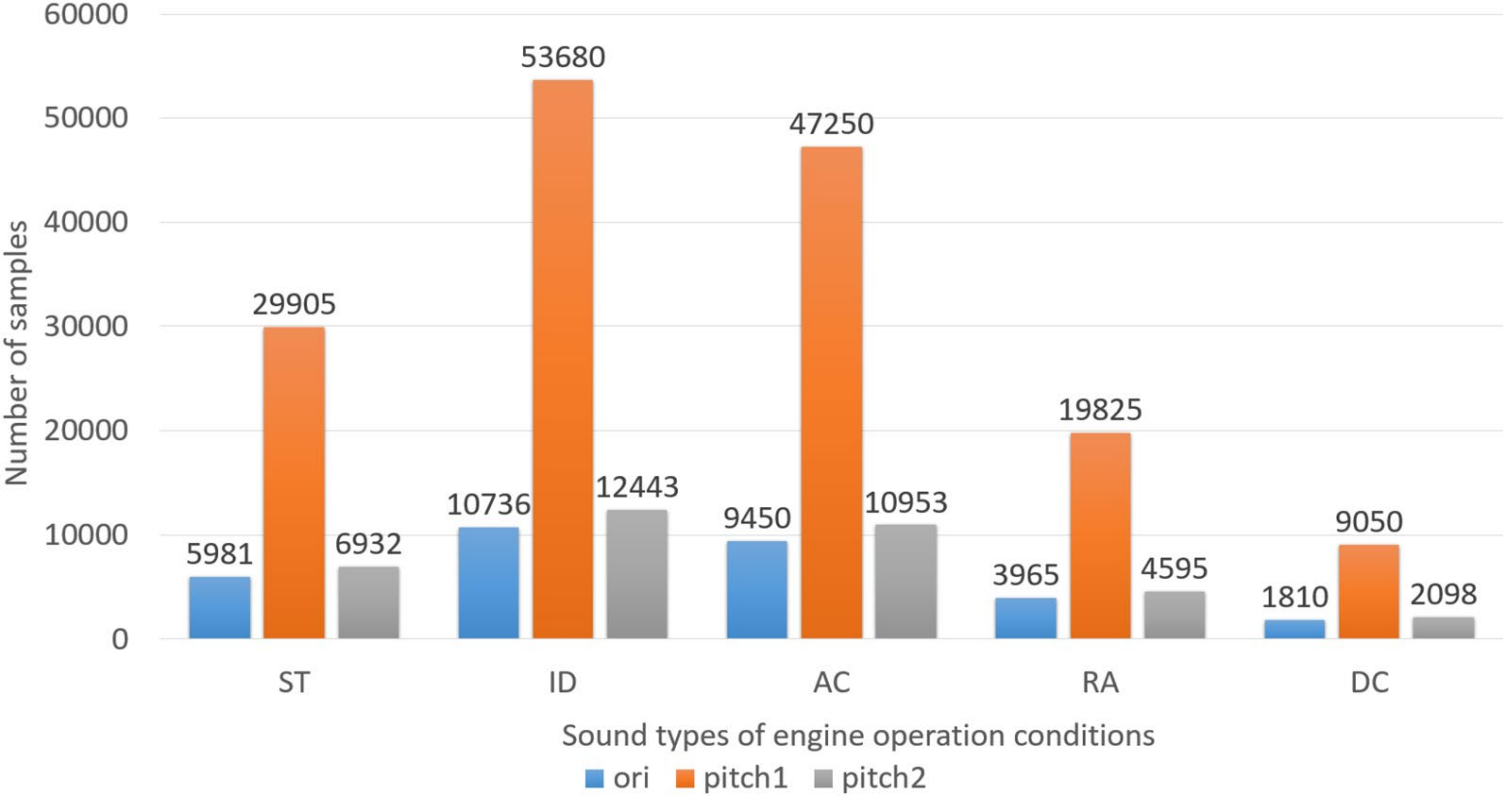
Comparison of sample numbers of engine sound dataset enhanced by different methods. *ST* Starting, *ID* Idling, *AC* Accelerating, *RA* Rapidly accelerating, *DC* Decelerating.

 We verified the effectiveness of the pitch shift method in Sect. Experimental for data enhancement. The method is as follows. For each type of engine sound, the pitch is shifted upward by values of {− 3.5, − 2.5, 2.5, 3.5} (semitone) until it reaches the standard number of samples in the fold. Due to the small number of deceleration operating condition sound samples, a set of shift parameters {− 4.5, − 4, − 3, − 2, − 1.5, − 1, 1, 1.5, 2, 3, 4, 4.5} is added, followed by data enhancement for deceleration operating condition sound according to two sets of parameters. Figure 12 compares the data enhanced by different methods to the original data, where "ori" refers to the original dataset, "pitch1" to the dataset enhanced by the method of Salamon and Bello^5^, and "pitch2" to the dataset enhanced by the method proposed in this paper.

The accuracy rate for recognizing the engine sound before and after data enhancement is shown in Table 2. It can be seen that the highest accuracy rate for recognizing engine sound is achieved using the data enhancement method proposed in this paper. This shows that the method of using different pitch shift parameters for the operating condition data with different number of samples can improve the accuracy of engine sound recognition.

**Table 2:** Engine sound recognition accuracy before and after data enhancement.

Data set	Accuracy (%)
Original data	84.87 ± 0.40
Enhanced data using method of Salamon and Bello^5^	85.52 ± 0.45
Enhanced data using method of this study	86.53 ± 0.23

**Figure 13:**
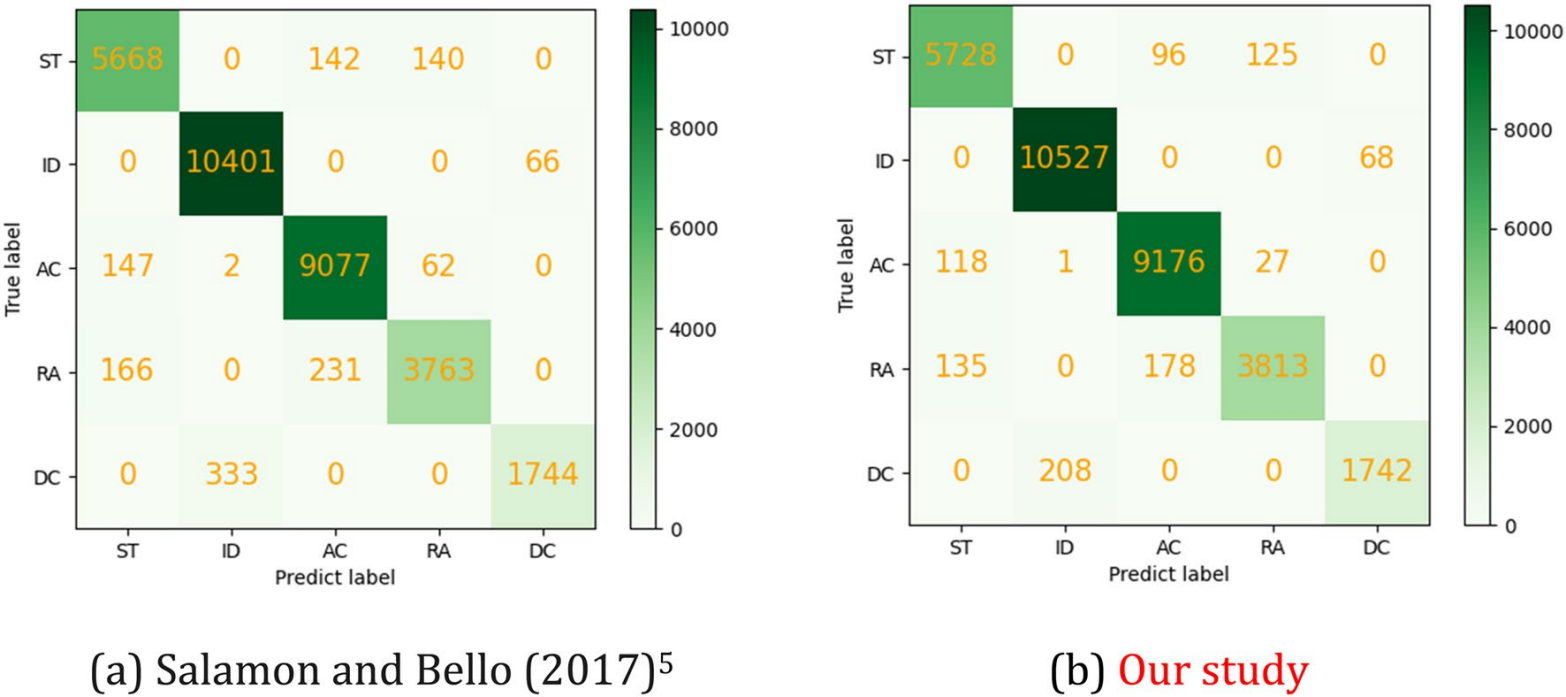
Confusion matrix of engine sound accuracy rate under different data enhancement methods.

Figure 13 shows the sound confusion matrix after the engine sound data are enhanced. The starting operating condition sound can be easily confused with the sounds of acceleration and rapid acceleration conditions, but this confusion is reduced compared to the method of Salamon and Bello^5^ when the data enhancement method proposed in this paper is used.

### Performance comparison experiment for ResNet with different structures

**Figure 14:**
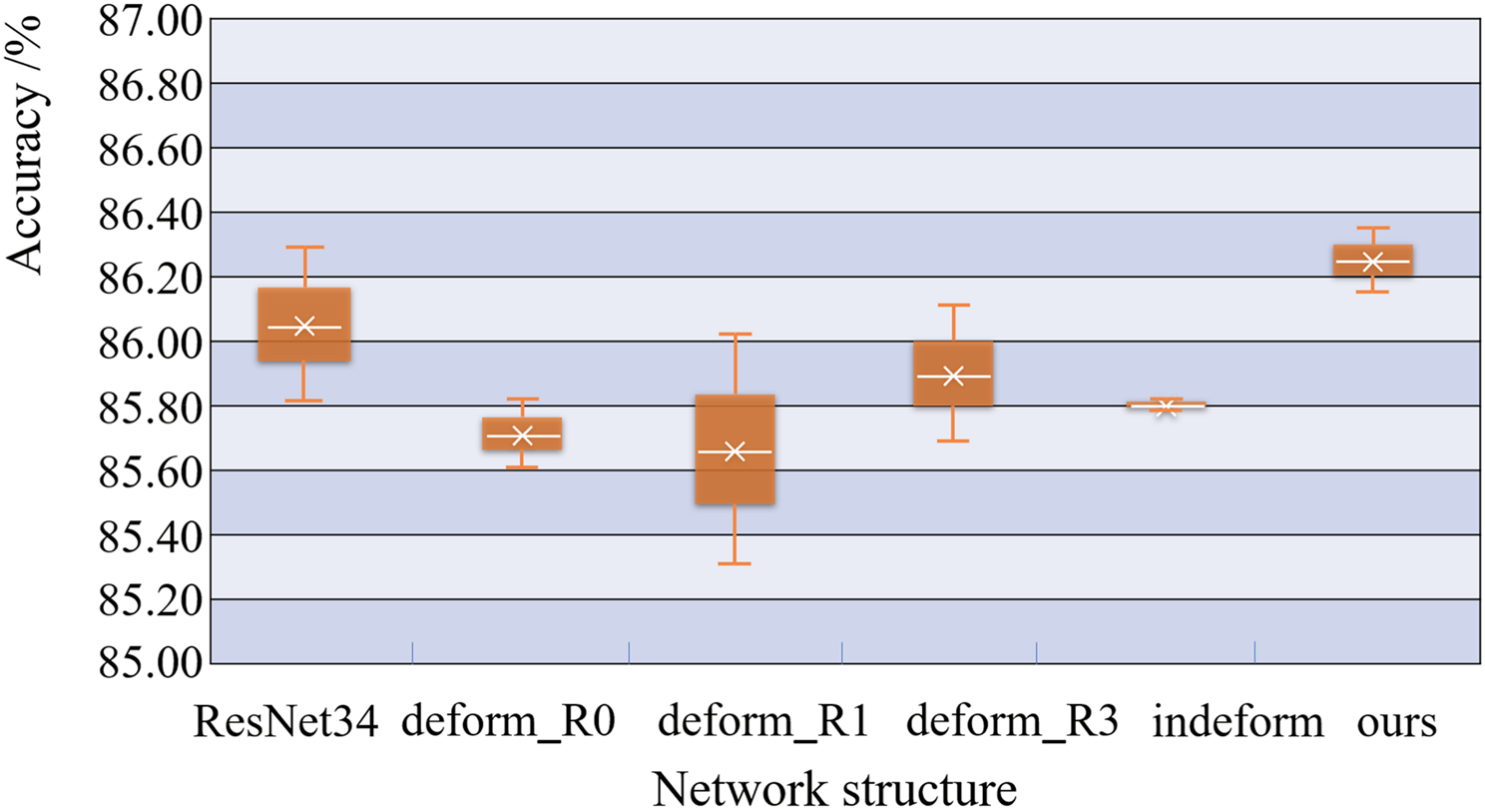
Classification accuracy of engine sounds by different network structures.

We compared the accuracy of our proposed method to that of other methods. As shown in Fig. 14, ResNet34 is a commonly used ResNet^13^; deform_R0 is a network in which three residual blocks of the top layer of ResNet34 are replaced by three DFMRB blocks; deform_R1 is a network in which every other ordinary residual block on the top layer of ResNet34 is replaced with DFMRB, using a total of three DFMRBs; and deform_R3 is a network in which every third ordinary residual block on the top layer of ResNet34 is replaced with DFMRB, using a total of three DFMRBs. "Ours" refers to the network used in the experiment of this section, whose structure is similar to that in Fig. 8. The difference is that the network used in this section does not include the SEB in Fig. 8 (its function is verified in the next section), i.e., we do not perform feature fusion. The designation "indeform" means that a deformable convolution residual block is used at the DFMRB position in the network of this paper^15^. The experimental input is a dataset enhanced according to the method in the section of Comparison Experiment of Data Enhancement Methods, and the experimental results are shown in Fig. 14. The " × " sign in the figure represents the average accuracy rate, and the height of the rectangle represents the degree of fluctuation of the accuracy rate.

The results in Fig. 14 show that, compared to ResNet34, the network used in this section has higher accuracy and less fluctuation. Compared to deform_R0, the average accuracy of the network in this paper increased, but the fluctuation of the accuracy rate is slightly higher. Compared to deform_R1 and deform_R3, the average accuracy of the network in this paper is larger, and the fluctuation is smaller. Compared to "indeform," the fluctuation of the network in this paper is slightly higher, and the accuracy rate is higher. Taken together, because the deformable feature map residual network proposed in this paper can focus the sampling in the region of interest of the feature map, it has the best performance in identifying engine sound.

### Comparison experiment on feature recalibration

We verified the effectiveness of the SEB feature recalibration module. The features acquired by the feature extraction module of the deformable feature map residual network were fused with MFCCs, and classification was performed after the fused features were recalibrated using SEB. The accuracy of the classification was compared to that when using fusion features without recalibration. Figure 8 shows the deformable feature map residual network, and Table 3 shows the experimental results.

**Table 3:** Accuracy of engine sound (%) before and after SEB feature recalibration.

Engine sound category	Accuracy	
	Before recalibration	After recalibration
Starting	82.70 ± 0.85	82.90 ± 0.57
Idling	73.65 ± 0.78	74.90 ± 0.00
Accelerating	98.27 ± 0.19	98.33 ± 0.18
Rapidly accelerating	99.94 ± 0.08	100.00 ± 0.00
Decelerating	75.15 ± 0.07	75.70 ± 0.06

In Table 3, we see that the accuracy rate for identifying engine sounds under different operating conditions using the recalibrated SEB feature is higher than when using SEB features without recalibration. Moreover, the standard deviation of the accuracy rate for recognizing sounds from various operating conditions is reduced. Because the feature recalibration module employed here uses two fully connected layers to fit different channel characteristics, we performed a weighted calculation for the channel features and the features extracted by the feature extraction module. This indicates that SEB recalibration can improve the accuracy of engine sound recognition under different operating conditions.

### Comparison experiment with current methods

We conducted comparison experiments with several typical methods using the AudioSet and VGGSound datasets, with results shown in Table 4. Because the accuracy rate published in today's open literature does not have a consistent format, we have unified them to contain two places after the decimal and listed them in Table 4. The proposed deformable feature map residual network has obvious advantages for engine operating condition recognition.

**Table 4:** Comparison of accuracy of engine sound under different operating conditions as obtained by different methods (%).

Method	Accuracy
Kemalkar et al.^2^	80.56
Salamon et al.^5^	82.22
Yang et al.^25^	82.88
Tien-Trien Le et al.^26^	83.24
Proposed method	84.28

## Discussion

We studied car engine sounds under five typical operating conditions and proposed a deformable feature map residual network for engine sound recognition. Without changing the positions of sampling points, DFMRB can pass the offset feature map information through a shortcut connect to the lower-level network, which can focus on sampling the region of interest in the input feature map and improve the feature extraction capability of the logarithmic Mel spectrogram for engine sounds under five operating conditions. The features extracted from the deformable feature map residual network are fused with MFCCs and recalibrated by SEB to further improve the engine sound recognition accuracy. It can be seen from the experimental results that, compared with the existing state-of-the-art methods, the method in this paper has the highest accuracy rate. This shows that the method in this paper has certain advantages and can be applied to recognize engine sounds in complex environments or under various engine operating conditions with an unknown signal-to-noise ratio. This provides a method of reference for forecasting automobile malfunctions using AI non-speech-recognition technology, especially for self-driving cars. In the deformable feature map residual network designed in this paper, the pixel offset distance is selected independently by the network within the range of the feature map. We did not study the optimal value of the offset distance, but subsequent research will determine the most suitable offset distance of the pixel.

## References

[1] Thomas DW, Wilkins BR (1972). The analysis of vehicle sounds for recognition. Pattern Recogn..

[2] Kemalkar, A. K., & Bairagi, V. K. Engine fault diagnosis using sound analysis, In *2016 International Conference on Automatic Control and Dynamic Optimization Techniques (ICACDOT). IEEE,* 943–946 (2016).

[3] de Oliveira LPR, Janssens K, Gajdatsy P (2009). Active sound quality control of engine induced cavity noise. Mech. Syst. Signal Pr..

[4] Wang YS, Ma QH, Zhu Q (2014). An intelligent approach for engine fault diagnosis based on Hilbert-Huang transform and support vector machine. Appl. Acoust..

[5] Salamon J, Bello JP (2017). Deep convolutional neural networks and data augmentation for environmental sound classification. IEEE Signal Proc. Let..

[6] Huang X, Huang H, Wu J (2020). Sound quality prediction and improving of vehicle interior noise based on deep convolutional neural networks. Expert Syst. Appl..

[7] Zhao L, Kang L, Yao S (2018). Research and application of acoustic emission signal processing technology. IEEE Access.

[8] Khan MS, Yu M, Feng P (2015). An unsupervised acoustic fall detection system using source separation for sound interference suppression. Signal Process.

[9] Krizhevsky A, Sutskever I, Hinton GE (2017). ImageNet classification with deep convolutional neural networks. Commun. ACM.

[10] Khare SK, Bajaj V (2020). Time-frequency representation and convolutional neural network-based emotion recognition. IEEE Trans. Neural Netw. Learn. Syst..

[11] Simonyan, K., & Zisserman, A. Very deep convolutional networks for large-scale image recognition, *arXiv preprint arXiv*:1409.1556, (2014).

[12] Khamparia A, Gupta D, Nguyen NG (2019). Sound classification using convolutional neural network and tensor deep stacking network. IEEE Access.

[13] He, K., Zhang, X., & Ren, S. Deep residual learning for image recognition, In *Proceedings of the IEEE conference on computer vision and pattern recognition. Los Alamitos: IEEE Computer Society Press*, 770–778 (2016).

[14] Wiatowski T, Bölcskei H (2017). A mathematical theory of deep convolutional neural networks for feature extraction. IEEE T. Inform. Theory.

[15] Dai, J. F., Qi, H. Z., & Xiong, Y. W. Deformable convolutional networks, In *Proceedings of the IEEE International Conference on Computer Vision. Los Alamitos: IEEE Computer Society Press,* 764–773 (2017).

[16] Leutnant V, Krueger A, Haeb-Umbach R (2013). A new observation model in the logarithmic mel power spectral domain for the automatic recognition of noisy reverberant speech. IEEE/ACM Trans. Audio Speech Lang. Process..

[17] Dennis J, Tran HD, Li HZ (2010). Spectrogram image feature for sound event classification in mismatched conditions. IEEE Signal Proc. Let..

[18] Hu, J., Shen, L., & Sun, G. Squeeze-and-excitation networks, In *Proceedings of the IEEE Conference On Computer Vision and Pattern Recognition. Los Alamitos: IEEE Computer Society Press*, 7132–7141 (2018).

[19] Liu, S. Y., & Deng, W. H. Very deep convolutional neural network based image classification using small training sample size, In *Proceedings of the 3rd IAPR Asian Conference on Pattern Recognition. Los Alamitos: IEEE Computer Society Press,* 730–734 (2015).

[20] Wang Y, Kwok J, Ni LM (2019). Generalizing from a few examples: A survey on few-shot learning. ACM Comput. Surv..

[21] Gemmeke, J. F., Ellis, D. P. W., & Freedman, D. Audio set: An ontology and human-labeled dataset for audio events, In *Proceedings of the IEEE International Conference on Acoustics, Speech and Signal Processing. Los Alamitos: IEEE Computer Society Press*, 776–780 (2017).

[22] Chen, H., Xie, W., & Vedaldi, A. Vggsound: A large-scale audio-visual dataset, In *ICASSP 2020–2020 IEEE International Conference on Acoustics, Speech and Signal Processing (ICASSP). IEEE*, 721–725 (2020).

[23] Ding, J. B., Ren, X. C., & Luo, R. X. An adaptive and Momental bound method for stochastic learning, (2019).

[24] Fuling L, Weihong Li, Weiguo G (2020). Deformable feature map residual network for urban sound recognition. J. Comput-Aid Des. Comput. Graph..

[25] Yang RY, Rai R (2019). Machine auscultation: enabling machine diagnostics using convolutional neural networks and large-scale machine audio data. Adv. Manuf..

[26] Le, T. T., Sagara, T., Kunioka, S. & Inose, S. A Fault Diagnosis Method for Fuel Injectors Using Machine Sound, In *2020 International Conference on Sensing, Diagnostics, Prognostics, and Control (SDPC)*, 30–34 (2020).

